# Flakka: New Dangerous Synthetic Cathinone on the Drug Scene

**DOI:** 10.3390/ijms21218185

**Published:** 2020-10-31

**Authors:** Jiri Patocka, Bingshu Zhao, Wenda Wu, Blanka Klimova, Martin Valis, Eugenie Nepovimova, Kamil Kuca

**Affiliations:** 1 Department of Radiology, Toxicology and Civil Protection, Faculty of Health and Social Studies, University of South Bohemia, 37005 Ceske Budejovice, Czech Republic; toxicology@toxicology.cz; 2 Biomedical Research Centre, University Hospital, 50003 Hradec Kralove, Czech Republic; 3 MOE Joint International Research Laboratory of Animal Health and Food Safety, College of Veterinary Medicine, Nanjing Agricultural University, Nanjing 210095, China; bzwh@163.com; 4 Department of Chemistry, Faculty of Science, University of Hradec Kralove, 50003 Hradec Kralove, Czech Republic; evzenie.n@seznam.cz; 5 Department of Neurology of the Medical Faculty of Charles University and University Hospital in Hradec Kralove, Sokolska 581, 50005 Hradec Kralove, Czech Republic; blanka.klimova@uhk.cz (B.K.); martin.valis@fnhk.cz (M.V.)

**Keywords:** Flakka, bath salts, synthetic cathinone, α-pyrrolidinopentiophenone

## Abstract

New psychoactive substances are being used as drugs and appear to be quite popular nowadays. Thanks to their specific properties, these drugs create inimitable experiences for intoxicated people. Synthetic cathinones are the most common compounds in these new drugs. Among them, α-pyrrolidopentadione (α-PVP), or “Flakka” (street name), is one of the most famous cathinone-designed drugs. Similar to other synthetic cathinone drugs, α-PVP can effectively inhibit norepinephrine and dopamine transmitters. The adverse reactions of α-PVP mainly include mania, tachycardia, and hallucinations. An increasing number of people are being admitted to emergency wards due to the consequences of their use. This work mainly summarizes the history, synthesis, pharmacology, toxicology, structure–activity relationship, metabolism, clinical process and health risks, poisoning and death, forensic toxicology, and legal status of α-PVP. We hope this review will help bring more attention to the exploration of this substance in order to raise awareness of its negative impacts on humans.

## 1. Introduction

Currently, drug abuse is a significant issue in the area of public health and, more importantly, among recreational users of the so-called “designer drugs”, “legal proceeds”, or “research chemicals”. There have been about 540 various drugs categorized as novel psychoactive substances since 2014 [1]. In addition, their number seems to continuously increase [2]. In the past few years, so-called “bath salts” have emerged. Bath salts are usually white or brown crystal-like powder, available for sale in small plastic bags marked “Not for Human Consumption”. They are also known as “plant food”, “phone screen cleaner”, or “jewelry cleaner” [3]. These names do not reflect the product properties; they are just a cover for the drug council or the local police. Therefore, these would-be products have nothing in common with actual “bath salts” or Epsom salts, which are developed from a mineral mixture of magnesium and sulphate, and which people put in bath water to help them relieve stress and relax muscles.

Bath salt is a name that has been created for a group of drugs that possesses one or more synthetic chemicals related to cathinone, a stimulant similar to amphetamines that is found in the plant khat (*Catha edulis*). Chemically, bath salts are derivatives of β-phenyl-ethylamine (PEA), including methamphetamine (N-methyl-1-phenylpropan-2-amine) and MDMA (3,4-methylendioxy-N-methylamfetamin, Ecstasy, or Molly). Compounds developed from cathinone (2-Amino-1-phenyl-1-propanone, benzoylethanamine, or β-keto-amphetamine) detected in PEA derivatives involve methylenedioxypyrovalerone (3,4-methylenedioxypyrovalerone, MDPV), 4-methylephedrone, mephedrone (2-methylamino-1-(4-methylphenyl)propan-1-one, 4-methyl-methcathinone, 4-MMC, Drone, Meph, or Meow Meow), methylone (3,4-methylenedioxy-N-methylcathinone, MDMC, bk-MDMA, M1), as well as many others. The range of chemical structures of these compounds is very broad and constantly changing.

Improper use of PEA derivatives or synthetic cathinone can be very dangerous [4]. Their use sometimes causes severe poisoning and considerably affects human health. Usually, people will be more anxious and aggressive after using synthetic cathinones, and they will exhibit aggressive behavior at high doses (70–100 ng/mL) [5]. In some cases, these substances have caused death [6,7,8]. Abuse of synthetic cathinones still represents a serious public health issue. Synthetic cathinone abuse mainly leads to sympathomimetic toxicity, which is mostly manifested as mania, tachycardia, high blood pressure, and occasionally hallucinations, hyponatremia, chest pain, and nausea. In severe cases it may cause epilepsy, severe peripheral organ damage, and rhabdomyolysis [9]. Data are only available from users who experience these possible hazardous consequences [10]. The purpose of this study is to review expert information about these dangerous synthetic drugs with respect to their chemistry, synthesis, metabolism, pharmacology, and toxicology.

## 2. Synthetic Cathinones History

Synthetic cathinones (e.g., mephedrone) were first synthesized in the 1920s and have chemical properties analogous to cathinones [11]. However, the term cathinone only appeared 40 years ago, and the term synthetic cathinone is even more recent [12]. Until recently, they had almost been forgotten. However, due to legal gaps, underground chemists started to exploit them in designer drugs [13,14,15]. Ten years ago, cathinone use began to increase again. Originally, they started to spread in Great Britain, then in other parts of Europe, and eventually in the United States [16], where medicines sold as PEA derivatives appeared in 2010 and were reported by US toxicological centers [14]. There has been a dramatic increase in the use of cathinones [17,18]. The U.K. National Poisons Information Service has collected telephone reports on the toxicity of mephedrone and other cathinone-related recreational drugs used in the UK. Before 2009, there had been no reports about these compounds in Great Britain. Between 2009 and 2010, there were 188 telephone reports related to cathinone, and most of them (157 calls) were related to mephedrone. Nevertheless, after 2010, the number of calls related to cathinone was equal to that of both MDMA (“Ecstasy”) and cocaine. Furthermore, there has been an alarming increase in the accessibility to these drugs, as well as increased exploitation and hospitalization in the United Kingdom and throughout Europe [19]. By mid-2015, α-PVP had caused 105 fatal intoxications in Europe [20]. In Europe, these drugs were mainly purchased on websites or in small packages as souvenirs.

**Figure 1:**
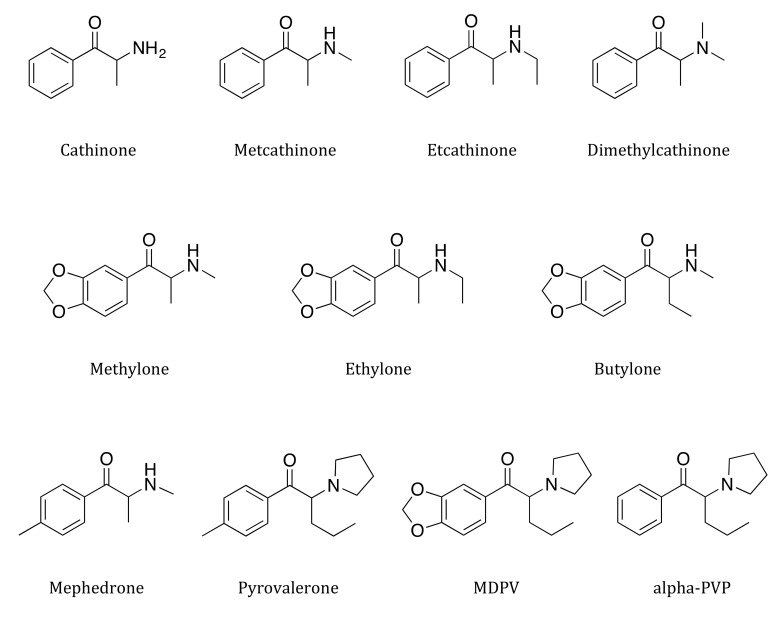
Chemical structures of some important synthetic cathinones.

Since then, there have been numerous new cases of designer drugs. These compounds are made to avoid illegal drug classification and this gives them the title of designer drugs [21,22]. In the short term, many synthetic cathinones have become popular, especially among younger generations [23]. Since reoccurring on the recreational drug market, there have been many reports in several countries of abuse, addiction, heavy drug abuse, and fatal intoxication associated with the use of synthetic cathinones [24,25,26]. During the last ten years, different synthetic cathinone derivatives have been detected on the market, which can be bought through the internet or in retail outlets. Synthetic cathinones are chemical derivatives of cathinones, natural monoamines, and they have been exploited for ages by the native inhabitants of the Horn of Africa and the Arabian Peninsula for their psychostimulatory features [27]. Most synthetic cathinones originate from China or India and are rapidly spreading around the world [28,29]. The chemical structures of important synthetic cathinones are summarized in Figure 1.

## 3. Flakka

A synthetic drug known on the streets as “Flakka” (α-pyrrolidinovalerophenone, α-PVP) has become popular in the United States. It is chemically similar to MDPV, also known as a bath salt, which was responsible for the increase in bizarre cases of intoxication and agitation in the United States several years ago [30]. Although people use α-PVP for their euphoric potential, symptoms can easily escalate into terrible delusions, paranoid psychosis, extreme agitation, and many other altered mental states. α-PVP causes a condition called agitated delirium, where there is an excessive influx of sympathetic activation [31]. This condition causes changes to the mental state, including bizarre behavior, anxiety, agitation, violence, confusion, myoclonus, and seizures [32,33]. Clinical signs of agitated delirium include tachycardia, hypertension, hyperthermia, diaphoresis, and mydriasis [34]. Although α-PVP is not a risk-free drug, this new synthetic Cathinone is beginning to dominate the drug arena in the US and Europe [35].

### 3.1. Synthesis

**Figure 2:**
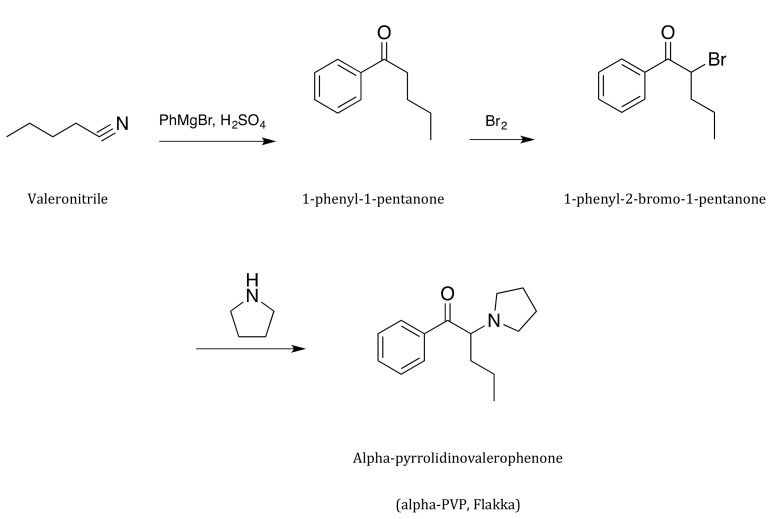
Flakka (α-pyrrolidinovalerophenone, α-PVP) synthesis.

Laboratory synthesis of α-PVP follows three steps. First, 1-phenyl1-pentanone is formed from the reaction of valeronitrile with phenylmagnesium bromide, with a subsequent acidic workup. It is then brominated to create the corresponding α-bromo ketone. This reacts with pyrrolidine to give α-PVP (51 % yield), which is eventually transformed in the hydrochloride salt [36,37] (Figure 2). α-PVP is used in its free base form or, rather, in the form of crystalline hydrochloride hydrate [38].

### 3.2. Pharmacology

Studies have shown [39,40] that, as one of the synthetic cathinones, α-PVP can increase extracellular neurotransmitter levels by inhibiting the monoamine transporters NET and DAT, corresponding to norepinephrine and dopamine, or by inhibiting the vesicular monoamine transporter (VMAT). α-PVP appears to have an effect analogous to MDPV, which is similar to a norepinephrine–dopamine reuptake inhibitor [41]. Metzler and coworkers have shown that α-PVPs have selective dopamine and norepinephrine receptor downtake activities, with little effect on serotonin (SERT) transport/reuptake [42]. The IC50 of this drug ranges between 12.8 and 205 nM for DAT, 4.2 and 20 nM for NET, and >10,000 to 30,000 nM for SERT in rats and humans, respectively [43].

α-PVP stimulates a considerable increase in locomotor performance, which appears much faster than the locomotor performance stimulated by methamphetamines [44]. This rise is stimulated by SCH23990 (50 µg/kg, i.p.), the D1 receptor antagonist, and sulpiride (50 mg/kg, i.m.), the D2 receptor antagonist. After α-PVP (25 mg/kg, p.o.) administration, the extracellular concentration of dopamine in the striatum increases. This suggests that α-PVP provokes dopamine delivery [45,46], resulting in an increased locomotor performance; thus it affects, at least in part, the activity of α-PVP on the Central Nervous System (CNS) by the D1 and D2 receptors [45,47].

There is increasing evidence that α-PVP has a cocaine-like stimulating effect. In fact, it is more effective than common psychostimulants such as cocaine and amphetamine [48,49]. Gannon and coworkers [48] used an absorption inhibition test in rat brain synaptosomes to directly compare the efficacy of MDPV, 3′,4′-Methylenedioxy-α-pyrrolidinobutiophenone (MDPBP), 3’,4’-Methylenedioxy-α-pyrrolidinopropiophenone (MDPPP), α-PVP, α-pyrrolidinopropiophenone (α-PPP), and cocaine on DAT, NET, and SERT. The results showed that α-PVP was in the same class and that α-PVP had the strongest selectivity for DAT and the lowest selectivity for SERT among the above-mentioned similar drugs. Collins and coworkers [49] used four adult male rhesus monkeys to study the self-administration of MDPV and α-PVP and directly compared the results with the effects of cocaine and methamphetamine. The results verified that synthetic cathinone MDPV and α-PVP could maintain a high level of response for an extended time and that they were more effective than cocaine or methamphetamine.

In order to explore whether synthetic cathinones have a direct myotoxicity, Zhou et al. [50] investigated the potential toxicological effects of synthetic cathinones on C2C12 myoblasts (a mouse skeletal muscle cell line). After exposing C2C12 myoblasts to α-PVP and other drugs for 1 h or 24 h, the cell membrane integrity, ATP content, mitochondrial oxygen consumption, and mitochondrial superoxide radical anion production were measured. The results showed that α-PVP consumes ATP, causes a loss of cell membrane integrity, and increases superoxide radical anion levels in C2C12 myoblasts in a concentration-dependent manner. In addition, as a pyrrolidone derivative, α-PVP also impairs basic and maximum cellular respiration, suggesting an abnormal mitochondrial function. Therefore, in addition to the effects on the sympathetic nervous system and vigorous muscle exercise, the direct effects of α-PVP on skeletal muscle mitochondria may lead to myotoxicity in susceptible cathinone users.

In a study with rodents, the acute administration of α-PVP at doses of 1–10 mg/kg by vapor or injection evoked a robust dose-dependent hyperlocomotion, likely involving enhanced signaling at dopamine D1 and D2 receptors [51]. Human psychopharmacological effects of α-PVP occur 10 min after a single dose (typically between 15 and 300 mg), peaking within 10–40 min and lasting for 2–3 h.

### 3.3. Toxicology

Kolesnikova and coworkers characterized the behavioral effects of α-PVP in adult zebrafish following acute (1, 5, 25, and 50 mg/L for 20 min) and chronic (1, 5, and 10 mg/L for seven days) treatments [43]. Overall, acute exposure to α-PVP evoked psychostimulant (but not anxiolytic-like) effects in this novel zebrafish tank test, with characteristic and stereotypic ‘side-to-side’ bottom swimming at 5, 25, and 50 mg/L. High-performance liquid chromatography/high-resolution mass spectrometry (HPLC/HRMS) analyses of zebrafish brains showed detectable levels of α-PVP following its acute administration, likely underlying the observed behavioral effects. The signal peaks for α-PVP in the brain were consistent with the systemic concentrations of the drug that were given. Although an acute two-day discontinuation after a chronic seven-day α-PVP administration at 1, 5, and 10 mg/L produced no effects, hypolocomotion and repeated withdrawal occurred after a seven-day chronic treatment, resembling the effects of some chronic psychostimulants. Collectively, these findings support zebrafish sensitivity to α-PVP and show some parallels with its effects in mammals. This study also suggests that aquatic models based on zebrafish can help to further examine the CNS effects evoked by α-PVP and to screen for related new synthetic psychoactive drugs. However, no experimental studies have been conducted to investigate the chronic impact of α-PVP on human health. In fact, knowledge on the long-term effects of α-PVP use in humans is lacking.

### 3.4. Structure–Activity Relationships

The strong pharmacological impact of α-PVP is likely caused by an inhibited uptake of dopamine by the dopamine transporter (DAT) [52]. Nevertheless, there is a lack of knowledge on how structural modifications to α-PVP affect DAT activity. To identify how biological activity depends on structure, 11 α-PVP analogs were formed, and their ability to inhibit 3H-dopamine and 3H-serotonin uptake by the serotonin transporter (SERT) was tested at 10 μM. All analogs acted as DAT reuptake inhibitors; however, the potency differed over a large range (> 1500 times). This potential was mainly related to the nature of the substituent, with larger and more numerous substituents giving a greater efficacy. Exchanging the pyrrolidine ring with piperidine reduces the activity 10-fold, while conformational restriction using aminotetralone leads to the least effective substance.

Nelson et al. [53] studied the stereoselective effects of α-PVP, and the results showed that the adverse effects of racemic α-PVP were mediated primarily by the S isomer. At the highest tested dose (6 mg/kg), the racemate induced an avoidance greater than the simple additive effects of S and R isomers (at 3 mg/kg), suggesting that while the R isomer may not induce a taste avoidance at this dose, it may interact synergistically with the S isomer in mediating the effects of the racemic mixture. These results were discussed in terms of similar effects to other behavioral and physiological endpoints reported with a number of psychostimulants, and they suggest that the enantiomers of α-PVP are an important variable in characterizing its behavioral effects.

Similarly, Schindler et al. [54] investigated the neurochemical, behavioral, and cardiovascular effects of racemic α-PVP and its enantiomers in male rats. The results showed that S-α-PVP is slightly more potent than racemic α-PVP, while R-α PVP is 10 to 20 times less effective in blocking dopamine and norepinephrine uptake. Racemic and S-α-PVP also increase the exercise capacity. When tested at the same dose, S-α-PVP produced a greater effect than racemic α-PVP. Racemic and S-α-PVP were self-administered by rats at a 0.03 mg/kg injection, whereas R-α-PVP was self-administered at a 10-fold higher dose. Dose–effect responses suggested that R-α-PVP was at least 30 times less potent than S-α-PVP. The results indicate that the neurochemical, behavioral, and cardiovascular effects of racemic α-PVP most likely reflect the effects of the S isomer.

### 3.5. Metabolism

An understanding of the metabolism of synthetic cathinones is valuable not only for antidoping and forensic purposes, but also to assist with the identification of metabolites that could potentially be further abused and require additional pharmacological evaluation. For example, several synthetic cathinones, including α-PVP, have been added to a list of banned substances at sporting events [55]. The metabolism of α-PVP and MDPV has been examined, but only the Phase I metabolism will be described here. Many of the metabolites can form sulfates or, more frequently, glucuronides, or other conjugates as Phase II metabolites. α-PVP metabolism has been explored mainly in rats [28,56]. Eleven metabolites of α-PVP were detected in rats, as well as in drug abusers’ urine samples. Phase I is recommended to involve metabolic steps, i.e., hydroxylation of the side chain followed by dehydrogenation to the relevant ketone, and hydroxylation of the 2-position of the pyrrolidine ring followed by dehydrogenation to the relevant lactam.

**Figure 3:**
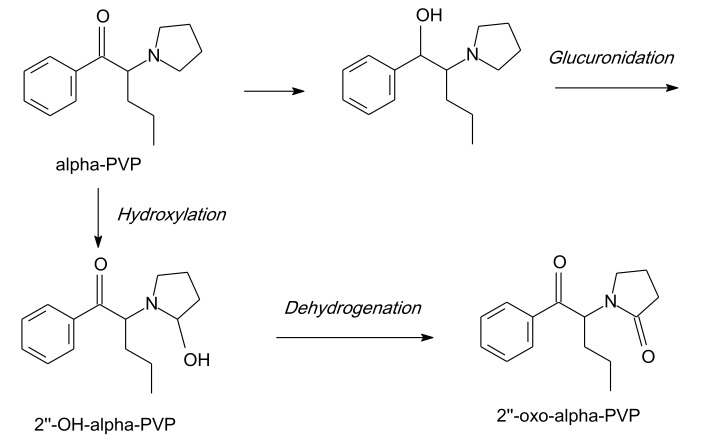
α-PVP metabolism.

Another route is to degrade the pyrrolidine ring to the corresponding primary amine, via hydroxylation of the phenyl ring, most likely to the 4-position, and to open the pyrrolidine ring to the corresponding carboxylic acid. Phase II metabolic steps include glucuronidation [57] (Figure 3). Nevertheless, there is a lack of knowledge regarding if these metabolites are pharmacologically functional.

When Shima et al. [37] analyzed 19 urine samples from α-PVP abusers, a decrease in the ketone set and oxidation of the pyrrolidine ring were the principal metabolic paths impacted. The 2’’-OH-α-PVP levels in urine were higher than the 2’’-oxo-α-PVP levels. This information revealed a interindividual dissimilarity in the main human metabolism of α-PVP, via a decrease of the ketone set or oxidation on position 2’’ of the pyrrolidine ring. The extremely high concentration of α-PVP and its metabolites (2’’-OH-α-PVP + 2’’-oxo-α-PVP) in urine (11,200 and 5300 ng/mL), and the comparatively excessive concentrations in the kidney (1580 and 972 ng/g, respectively), revealed that α-PVP was quickly excreted into urine through the kidney [58]. There have been no experimental studies on the chronic health impact of α-PVP and/or its metabolites in humans.

Thus, it is still necessary to further integrate human and animal models (including humans, rodents, and aquatic organisms) to understand the biological life span of α-PVP metabolites and whether there is any biological activity in major species.

### 3.6. Clinical Course and Health Risks

The public health risks connected with α-PVP depend on several factors: frequency and method of administration, accessibility and drug properties, the range of knowledge among users, and adverse health effects. There is a lack of relevant information on sporadic versus chronic use, which would help to prevent risks connected with α-PVP. The renewal and abuse of illicit drugs brings difficulties to clinicians in terms of screening, testing, and treatment, and it hinders different drug monitoring agencies from determining appropriate regulations.

α-PVP can be orally ingested, smoked, snorted, used parenterally, and also vaporized in e-cigarette devices. Vaporizing drugs in e-cigarettes is becoming a common method of administration for synthetic cathinones and classical stimulants. Heating during vaporization can expose the user to a cocktail of parent compounds and thermolytic degradants, which could lead to different toxicological and pharmacological effects compared to ingesting the parent compound alone via injection or nasal inhalation [59,60]. This latter route of administration leads to a rapid introduction into the blood stream, resulting in a high risk of overdose. α-PVP produces a variety of symptoms when consumed. The drug has been reported to cause depression, panic attacks, chest pains, paranoia, hallucinations, aggressive behavior, self-injury behaviors (including suicide), and prolonged psychosis [31]. The speed at which new psychoactive drugs appear is alarming. New structures have new properties and thus create unique behavioral characteristics during intoxication. It can be assumed that the tendency towards the use of new drugs will grow and that there will be an increasing number of cases in clinical practice. Doctors have to take this into consideration in order to provide appropriate treatments and minimize undesirable damage.

The findings reveal that α-PVP is exploited by recreational and problem drug users. The latter also take opioid and stimulant drugs. Heikman et al. [61] indicated that the frequent use of multiple drugs may be common in patients using α-PVP. About 0.8% of high school seniors in 2016/2017 were estimated to have used α-PVP in the past year [62]. Students whose parents had less than a high school education were at higher odds of use. α-PVP users report a high prevalence of use of other drugs, particularly synthetic cannabinoids (85.6%), ketamine (72.3%), marijuana (59.1%), and GHB (47.5%). α-PVP use is also associated with a higher number and frequency of using other drugs, with 51.7% using 4–12 other drugs and 22.4% using 4–12 other drugs > 6 times.

### 3.7. Intoxications and Fatalities

α-PVP poisoning was notably detected in Scandinavian countries, Denmark, and Iceland in 2012. In Finland, there were 162 cases of using various illegal drugs in 2012, confirmed by positive test results. Among them, the incidence of α-PVP poisoning was 4.9%. In Sweden, there were 255 cases using various illegal drugs, confirmed by positive test results. Among them, the incidence of α-PVP poisoning was 0.4%. Most cases involved other NPS and/or classic drugs [63]. From April 2013 to November 2015, there were 31 patients with α-PVP poisoning, of which 73% were known drug users [64]. All cases involved other NPS and/or classic drugs. α-pyrrolidinobutiophenone (α-PBP) and other pyrrolidone analogs are the most common other NPS drugs, and benzodiazepines are the most common CNS inhibitors. Of the 14 cases requiring monitoring in an intensive care unit, eight were judged to be severely poisoned. No deaths were reported.

### 3.8. Forensic Toxicology

In 2012, α-PVP was identified in the postmortem samples of a drowned victim at concentrations of 40 and 295 ng/mL in the blood and urine. In Finland, there were two fatal intoxications: suicide and accidental death. In the case of the suicide by doxepine poisoning, α-PVP was identified in the blood (70 ng/mL), and doxepine, citalopram, quetiapine, MDPV, buprenorphine, and temazepam were also identified. In the case of the accidental death due to multiple injuries, α-PVP (60 ng/mL), amphetamine, and ketamine were identified in the blood [65]. In addition, Richards–Waugh et al. [66] described three instances of α-PVP-related deaths of three adults aged 31, 35, and 51 years. Their toxicological analysis revealed blood concentrations of 29, 52, and 10 ng/mL. All three men exhibited typical signs of these drugs: aggressive behavior and suicidal inclination in the first case and seizures in the second case. The toxicological analysis also revealed the presence of sertraline, oxycodone, and 7-aminoclonazepam. In all three cases, α-PVP contributed to their death; however, it was not the main factor [66].

Similar fatal cases were also reported in Ohio [29]. Four women between 32 and 44 years, and two men aged 34 and 51 years, died of fatal intoxication. α-PVP was detected in their blood and urine postmortem. The occurrence of other drugs was also evidenced (morphine, codeine, hydrocodone, and 6-monoacetylmorphine) in the four cases, cocaine and its metabolites in one case, and synthetic cathinones (MDPV, pentylone, and methedrone) in three cases. Quetiapine was detected in one case, and amitriptyline, citalopram, venlafaxine, and norvenlafaxine were detected in three cases [29]. In one of the first reports of fatal α-PVP poisoning in humans, where α-PVP was the only cause of death described [67], the heart blood concentration of the drug was 486 ng/mL.

In Japan, a fatal case of sudden death due to cardiac failure of a 41-year-old man who had self-administered α-PVP was reported in 2014 [68]. The toxicological analysis revealed other substances as well, but α-PVP was detected (411 ng/mL) in postmortem blood samples and in blood samples from the right and left ventricles and femoral vein [58]. α-PVP levels were identified at concentrations of 597, 635, and 580 ng/mL in the blood from the right and left ventricles and femoral vein, respectively [69]. Α-PVP was also evidenced in the urine. Other cases of α-PVP poisoning are on the rise. In the USA, there were over 300 cases of α-PVP exploitation in the first three months of 2015 [70], and 18 fatal deaths were reported in only one south Florida county [71]. A brief review of the previously reported human α-PVP poisoning cases is summarized in Table 1.

### 3.9. Legal Status of α-PVP

Currently, α-PVP is a strictly controlled substance, and its use has been banned in the United States and other countries/regions around the world. In March 2014 [77], the DEA temporarily included 10 types of synthetic cathinones, such as α-PVP, in the Schedules of Controlled Substances, and they extended this control period in March 2016 [78]. In the final rules promulgated by DEA in March 2017 [79], α-PVP was listed as a Schedule I drug.

In 2015, the European Monitoring Center for Drugs and Drug Addiction (EMCDDA) conducted a risk assessment for α-PVP. According to the results, on June 27, 2016, the European Council decided to enact control measures for α-PVP that applied to all member states. Currently, Estonia, Finland, France, Germany, Hungary, Ireland, Latvia, Lithuania, Poland, Romania, Slovenia, Sweden, the United Kingdom, Turkey, Norway, and the Czech Republic all prohibit the use of α-PVP [20].

In October 2015, α-PVP was classified as a controlled substance in China [80].

In Australia, α-PVP is a banned substance according to Schedule 9 of the Poison Standard (July 2016) [81]. Schedule 9 substances refer to substances that may be abused. Unless required for medical or scientific research or analysis, or for teaching or training purposes, the law should prohibit their manufacture, possession, sale, or use.

## 4. Conclusions

The use of khat and synthetic cathinones has increased worldwide during the past several decades. Apart from resulting in CNS stimulation and euphoria, these compounds exhibit other negative impacts, mostly resembling those of amphetamine. In the course of the last four years, Flakka (α-PVP) has penetrated markets all over the world, especially the US, European states, Australia, and Japan. This increase is mainly due to its simple preparation and low price. The growing number of seizures, intoxications, and deaths connected with α-PVP exploitation, and the lack of a relevant antidote for cathinone exposure, requires further investigation among toxicologists. In addition, national governments should take relevant measures in order to control the use of this novel dangerous drug. Future research should focus on the development of analytical methods for rapid identification in both live and dead specimens.

## Figures and Tables

**Table 1:** Fatalities after α-PVP application published in medical literature.

Country Year	Number Intoxicated/Death	α-PVP in Blood	Other Drugs Found in the Blood	Remarks Information	Reference
Finland 2012	1/1	70 ng/mL	doxepine, citalopram, quetiapine, buprenorphine temazepam.	suicide	[65]
Finland 2014	1/1	60 ng/mL	amphetamine, ketamine.		[65]
United States 2013	3/3	10 ng/mL 29 ng/mL 52 ng/mL	pentedrone sertraline, 7-amino-lonazepam, oxycodone, THC.	deaths of three men aged 31, 35, and 51 years.	[66]
United States 2013	6/6	only qualitative estimation	morphine, codeine, hydrocodone, 6-monoacetylmorphine	deaths of four women between 32 and 44 years, and two men aged 34 and 51 years.	[29]
United States 2012-2015	21/3	50-90 ng/mL	clonazepam, diazepam, oxycodone, THC, alprazolam, oxazepam, tamazepam.		[72]
Japan 2013	1/1	486 ng/mL	no other drugs.		[67]
Japan 2014	2/2	411 ng/mL 579 ng/mL	no other drugs.		[58,68]
Australia 2014	1/1	only qualitative estimation	no other drugs.	cardiac arrest	[73]
Poland 2015	1/1	185 ng/ml	pentedrone.		[74]
Poland 2016	1/1	174 ng/ml	no other drugs.	technique revealed the presence of α-PVP in the following concentrations: urine, 401 ng/mL; brain, 292 ng/g; liver, 190 ng/g; kidney, 122 ng/g; gastric content, 606 ng/g. cardiac arrest secondary to intoxication with alpha-PVP was determined as the direct cause of the patient’s death.	[75]
Finland 2020	1/1	800 ng/ml	amphetamine, buprenorphine, benzodiazepines.	sudden death of a middle-aged man while having a sauna.	[76]
